# *Raman2imzML* converts Raman imaging data into the standard mass spectrometry imaging format

**DOI:** 10.1186/s12859-020-03789-8

**Published:** 2020-10-09

**Authors:** Stefania Alexandra Iakab, Lluc Sementé, María García-Altares, Xavier Correig, Pere Ràfols

**Affiliations:** 1 Department of Electronic Engineering, Rovira i Virgili University, 43007 Tarragona, Spain; 2 Spanish Biomedical Research Centre in Diabetes and Associated Metabolic Disorders (CIBERDEM), 28029 Madrid, Spain; 3 Institut d’Investigació Sanitària Pere Virgili, Tarragona, Spain

**Keywords:** Converter, Raman imaging, imzML, R, Mass spectrometry imaging

## Abstract

**Background:**

Multimodal imaging that combines mass spectrometry imaging (MSI) with Raman imaging is a rapidly developing multidisciplinary analytical method used by a growing number of research groups. Computational tools that can visualize and aid the analysis of datasets by both techniques are in demand.

**Results:**

*Raman2imzML* was developed as an open-source converter that transforms Raman imaging data into imzML, a standardized common data format created and adopted by the mass spectrometry community. We successfully converted Raman datasets to imzML and visualized Raman images using open-source software designed for MSI applications.

**Conclusion:**

*Raman2imzML* enables both MSI and Raman images to be visualized using the same file format and the same software for a straightforward exploratory imaging analysis.

## Background

In recent years, mass spectrometry imaging (MSI) has become an important analytical technique because of its capacity to spatially localize a wide range of biomolecules from plant, animal and human tissues [1]. The main advantage of MSI is its high specificity, which makes it possible to identify endogenous and exogenous compounds such as metabolites, lipids, peptides and proteins. Consequently, a considerable number of advanced data analysis tools have emerged as proprietary or open source software, with a tendency towards data format standardization [2].

However, the most common MSI instruments and sample preparation protocols have difficulty in acquiring high spatial resolution images. The spatial resolution of current acquisitions is limited to a few micrometres, which prevents the detailed molecular characterization at the micron scale so necessary for the study of microorganisms and cells [3]. Thus, it was suggested that the molecular images produced by MSI could be combined with images obtained by other high spatial resolution techniques. Several studies proposed correlating MSI with optical or fluorescence images [4–6] or with molecular images of spectroscopic techniques such as Fourier Transformed-Infrared Spectroscopy (FTIR) [7, 8] and Raman Spectroscopy imaging [9–17]. The combination of MSI and Raman imaging is becoming increasingly popular for exploring biological tissues because Raman is a non-destructive label-free technique which characterizes tissues at a submicron lateral resolution. Moreover, Raman imaging provides MSI proteomic studies with complimentary information about the chemical composition of the sample, such as lipid-to-protein ratio or changes in lipid content [10].

Nonetheless, collecting molecular information for sample characterization with two analytical instruments means that there are two different data formats, which need two different software to visualize and analyse the two datasets. A unified data format, common to all spectral imaging techniques, would benefit and promote the development of multimodal imaging applications. So far, only a few studies have visualized and analysed datasets using techniques such as spectroscopic, mass spectrometric, and X-ray diffraction data within the same software [18–20]. However, the data format supported in these cases was common text (.txt) which is not suitable for MSI datasets because it cannot encode a complete MSI dataset in a text file because of its large data size. The standard data format adopted by the MSI community to exchange and process data is imzML [21]. Currently instrument manufacturers and the scientific community are not using a standard file format for Raman imaging data. Due to the similarities between MSI and Raman imaging data structure, we suggest adapting Raman imaging datasets to the standardized open format imzML. In this way, MSI and Raman imaging data can be explored with the same software, so that data from different imaging experiments can be visualized more straightforwardly.

Here, we present *Raman2imzML,* an open-source data converter distributed as an R package which converts Raman data acquired with Renishaw and WITec instruments to imzML. We converted Raman imaging datasets collected from mouse brain tissue, and then visualized them with commonly used MSI software tools. The *Raman2imzML* package together with its documentation is available online (github.com).

## Implementation

**Fig. 1:**
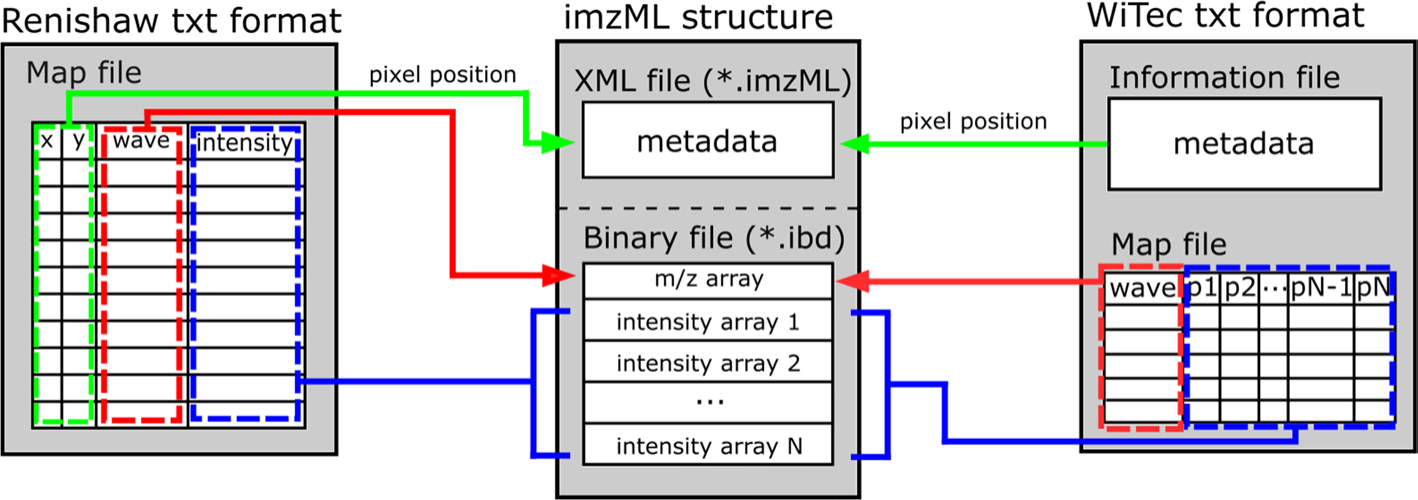
Data conversion from Raman *.txt files (exported from datasets collected with Renishaw and WITec spectrometers) into the imzML format. Coloured arrows indicate how and where the information is stored in the imzML format

*Raman2imzML* converts text files (.txt) exported from imaging data acquired using Renishaw and WITec Raman instruments to imzML format (Fig. 1). To convert the Raman data to imzML, the Raman text file (.txt) is parsed to extract the imaging information: number of pixels, pixel coordinates and number of data points in each spectrum. Then, the imaging information is used to calculate the binary offsets according to the imzML format specification and stored in an intermediate data structure. Immediately, the binary part of the imzML format is written by directly transferring each Raman spectrum to binary stream encoded as 32-bit floating-point numbers. Finally, the *Raman2imzML* converter uses the imzML parser in the rMSI package [22] to write the XML part of the imzML using the imaging information (metadata) stored in the intermediate data structure. The *Raman2imzML* converter is distributed as an R package and includes a converter function for each instrument manufacturer to simplify user access. A Raman2imzML R project that includes a markdown and a set of example measurements from Renishaw and WITec to test the package is available in Additional files 1 and 2.

The imzML format was designed to store mass spectrometry data, so it has some limitations when it is used for Raman data. Several specific words defined in the imzML ontology do not suit Raman experiments or do not exist. For example, “Raman Shift” units or diffraction grating definitions (cm^−1^) are nonexistent in the imzML format. Therefore, after the conversion, only numeric imaging information such as pixel position, intensity values and Raman shift axis values are preserved. It should also be pointed out that some information (integration time, excitation wavelength, etc.) is not preserved by the converter, as it is lost when the data is exported to text. Nevertheless, we suggest that the imzML specification be extended to handle imaging modalities other than MSI. This will enable future versions of *Raman2imzML* to perform data conversion without losing any information.

## Results and discussion

**Fig. 2:**
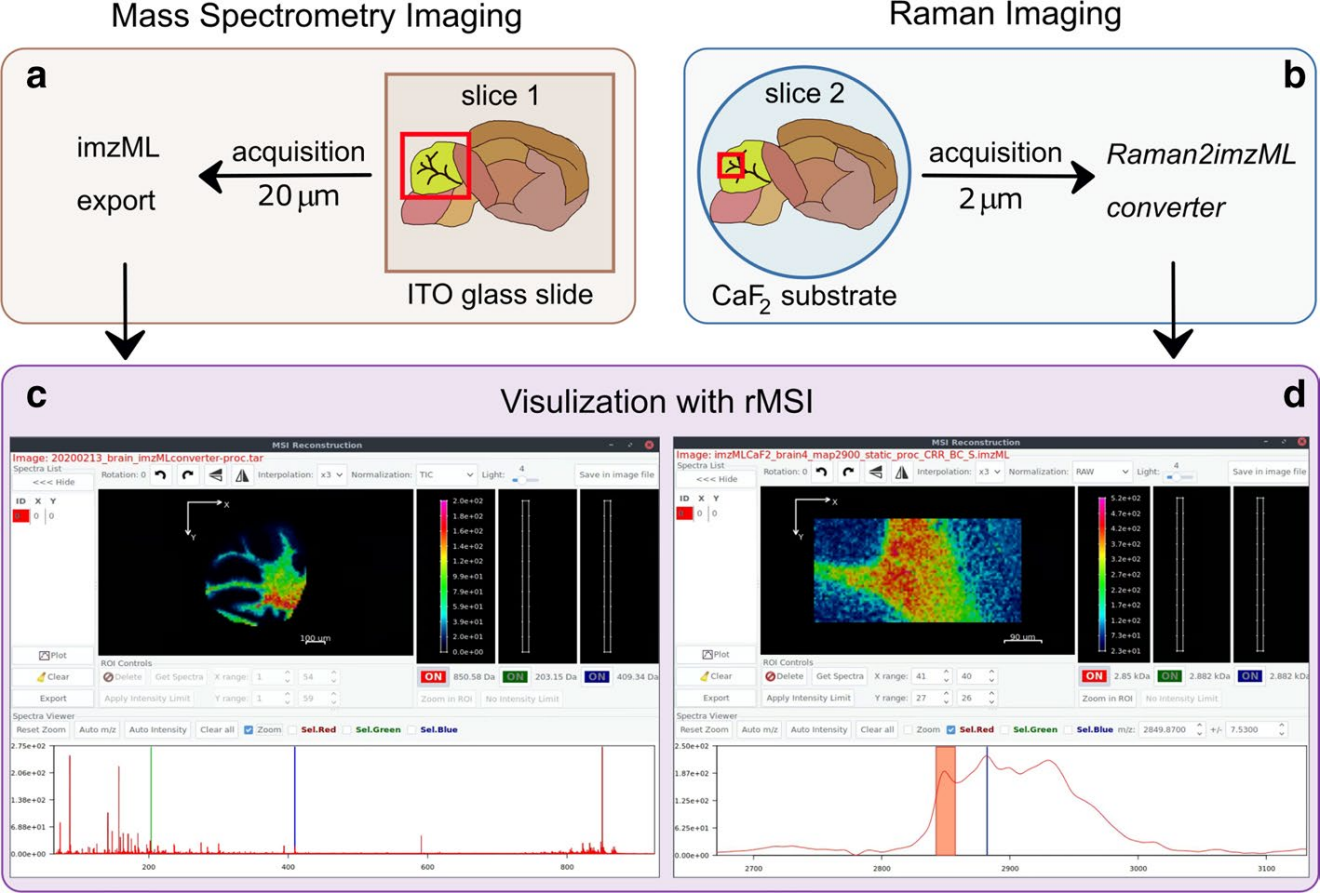
Imaging mouse brain sections: MSI (**a**) and Raman imaging (**b**) sample preparation steps and visualization within the same open-source software (rMSI) of each dataset (**c** and **d**); rMSI: R package for MSI data handling and visualization, Copyright© 2014 Pere Rafols Soler

**Fig. 3:**
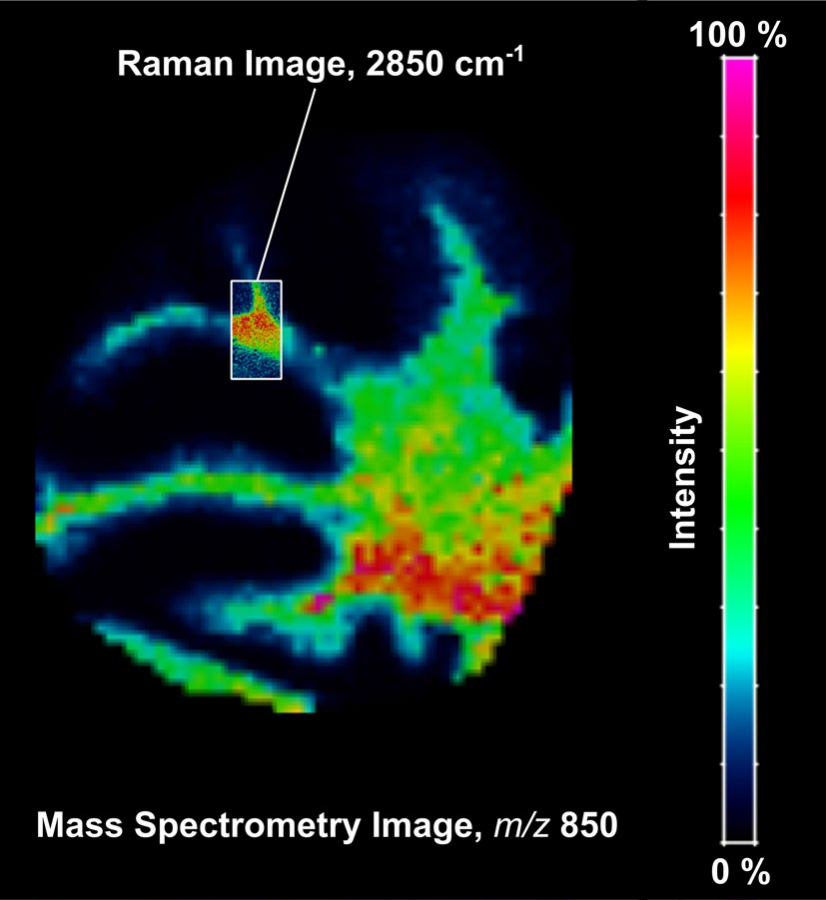
Merged molecular images collected from MSI (20 μm lateral resolution) and Raman (2 μm lateral resolution). Molecular images are the same as Fig. 2

Figure 2 illustrates how images produced by MSI and Raman imaging can be visualized and explored using the same software. First, we used laser desorption/ionization (LDI) MSI and Raman to acquire molecular images from consecutive histological sections of a fresh-frozen mouse brain. For MSI (Fig. 2a), we placed the tissue section onto an ITO-coated glass slide and covered the surface of the tissue with a thin Au nanolayer by sputtering [23]. For Raman measurements (Fig. 2b), we placed the consecutive tissue slice onto a CaF_2_ slide. Images were acquired using MALDI TOF/TOF UltrafleXtreme (50–1200 Da mass range, positive reflectron mode, large laser spot size, 500 shots per pixel, 20 μm lateral resolution) for MSI, and Renishaw (633 nm excitation wavelength, 50x objective, 2 s integration time, 50% laser power, 2 μm step) for Raman imaging. To explore both imaging datasets, we exported the MSI data using Bruker’s software flexImaging™ directly into imzML, and converted the Raman text data using *Raman2imzML* into the imzML format. Finally, we imported both imzML files independently, and visualized the molecular images using the rMSI package [22] although other MSI software can be used for data exploration: for example, open-source options such as Datacube Explorer [24], CARDINAL [25], MSiReader [26] and msiQuant [27] and commercial options such as SCiLS Lab and MALDIVision. For MSI (Fig. 2c), we showed the spatial distribution of ion *m/z* 850 (putatively annotated as a glycerophospholipid—phosphatidylcholine (38:3), experimental *m/z* 850.58 as [M + K]^+^ adduct, mass error 9 ppm) [28] on the whole mouse cerebellum. For Raman (Fig. 2d) we chose to illustrate the band at 2850 cm^−1^ Raman shift, which is specific for lipids [29] on one specific nerve tract in the cerebellum. The possible merger of the two molecular images (Fig. 2c and d) is shown in Fig. 3. The high spatial resolution image from Raman clearly enhances the low-resolution image obtained with MSI. When co-registration strategies are used together with multimodal imaging techniques it can accurately coordinate the relationship between pixel position and information between the two images. This way Raman2imzML could also enable hyperspectral data analysis of Raman and MSI datasets within the same software.

## Conclusion

The *Raman2imzML* converter was created so that the same software could be used to visualize different imaging datasets, and in particular to explore Raman images using MSI software tools. We demonstrated that the similarities between Raman imaging and MSI data structures favour the use of the same data visualization software. Therefore, we recommend that a comprehensive multimodal strategy be created to facilitate the combination of spectral imaging techniques such as Raman and MSI. We propose that imzML be used as a template for a standardized file format for Raman imaging and other spectral imaging techniques, and that specific Raman ontology be included in future imzML format iterations.

## Availability and requirements

- Project name: Raman2imzML.
- Project home page: https://github.com/LlucSF/Raman2imzML.
- Operating system(s): Platform independent.
- Other requirements: R, Rtools, RStudio.
- Programming language: R and C++.
- License: GNU GPL-3.0.
- Any restrictions to use by non-academics: none.
- The datasets generated and/or analysed during the current study are available in the GitHub repository, https://github.com/LlucSF/Raman2imzML. The associated additional files contains the Raman2imzML R project that includes a markdown and a set of example measurements to test this package.

## Supplementary information

**Additional file 1.** Example datasets collected with Renishaw and WITec Raman instruments as common text files.

**Additional file 2.** Markdown for using the Raman2imzML package.

## Abbreviations

MSI: Mass spectrometry imaging
FTIR: Fourier transformed-infrared spectroscopy
LDI-MSI: Laser desorption/ionization mass spectrometry imaging
